# Monte Carlo Simulation of Protein Adsorption on Energetically Heterogeneous Surfaces

**DOI:** 10.1155/2014/278098

**Published:** 2014-07-16

**Authors:** Panu Danwanichakul

**Affiliations:** Department of Chemical Engineering, Faculty of Engineering, Thammasat University, 99 Moo 18 Phaholyothin Road, Khlong-Luang, Pathum Thani 12120, Thailand

## Abstract

The modified triangular-well potential model was applied to incorporate the effect of surface energy on the adsorption of particles or proteins on energetically heterogeneous surfaces. The method is convenient in simulating the adsorption on heterogeneous surface of which different region possesses different free energy. Spherical particles with attractive forces were added on the surface and underwent surface diffusion before they were quenched in place. It was seen that the ratio of surface energies of two regions had to be greater than 10 in order to simulate the adsorption in which the particles were selectively adsorbed on a favorable area. At a fixed ratio of surface energies, the obtained structures were similar. If the ratio was less than 10, the probability of adsorption on any site on the surface was not much different so the adsorption would be homogeneous adsorption. The method, thus, could be applied widely to simulate the adsorption of various conditions.

## 1. Introduction

The structures and properties of monolayer films generated by deposition of colloidal particles or bioparticles such as proteins on solid surfaces are of importance in many applications including coating and biosensor. A plastic surface was coated with a biocompatible material so that it could be safely used as an implant in human body [1]. For example, polyethylene glycol (PEG) was coated on titanium surface to prevent adsorption of proteins in blood [2] and it could also be adsorbed on PANCMA membrane to increase biocompatibility [3]. In addition, adsorption or immobilization of DNA molecules on a biochip for checking genetic diseases has gained much attraction [4]. Adsorption of such molecules or particles on a surface depends on detailed mechanism including molecular transport from solution or suspension to the surface, surface diffusion of molecules under the effect of previously adsorbed molecules, reversible or irreversible attachment to the surface, and desorption of molecules from the surface when adsorption is reversible. The reversible adsorption could finally reach equilibrium at which point the adsorption rate and desorption rate are dynamically equal and the equilibrium ordered structure of the film is obtained. However, adsorption of bioparticles such as proteins is normally classified as irreversible adsorption in which the attractive forces among adsorbed molecules and the surface are so strong that surface diffusion eventually stops and desorption is not going to happen.

Feder and Giaever [5] reported that monolayer structures of adsorbed ferritin on carbon surface could be explained by a model of irreversible adsorption, namely, random sequential adsorption (RSA), where no surface diffusion and desorption are at all involved. Onoda and Liniger [6] also found that the configurations of polystyrene spheres on a glass surface finally reached the jamming limit of RSA. However, there have been many indirect and direct evidences showing that among the immobile particles on the surface there exists mobile fraction of particles [7, 8]. The change in conformation or orientation of the adsorbed proteins [9], the composition of the bulk solution [10], and nature of the surface [11] have been reported to be responsible for lateral mobility and for adsorption irreversibility. A new model called sequential quenching (SQ) was then proposed to embody the step of lateral mobility on the surface [12]. The SQ model may be regarded as an extended version of RSA, in which the surface diffusion step follows the arrival of newly added particles. It is assumed that there is possible presence of strong localized interactions between adsorbate, that is, colloidal particles or proteins, and substrate, which may be considered as a (first order) chemical reaction, and that the activation energy for desorption is much higher than those for other processes.

Based on models proposed for such adsorption, the maximum surface coverage of the monolayer could be obtained from simulations together with the proposed kinetic model of deposition. The structure of a monolayer film could also be obtained and represented by the radial distribution function which illustrates the probability of finding a particle at any radial position from the center particle. Some works [13–15] were focused on the adsorption of particles on homogeneous surfaces of which any position was assumed to possess uniform surface energy, thereby, particles being attached on the surface at random. In real applications, however, adsorption occurs on surfaces upon which some regions may be modified chemically or physically by coating with a polymer, a surfactant, or a chemical coupling agent such as silane which could change the surface charges [16, 17]. The surface heterogeneity then plays a significant role in colloidal and protein adsorption. Many evidences should be raised as examples. Unlike what happened in adsorption on homogeneous glass bead surface, the adsorption of heavy metal ions at different position on oxide surfaces depended on different interaction between surface and adsorbed ions resulting in different charge distribution on the surface [18]. Another obvious example is DNA chips where the probe strands are first immobilized which generates heterogeneity on the surface and the hybridization happens when the target strands find their probe pairs.

The efficiency of immobilization of probe strands, which is irreversible adsorption, could be improved by surface modification methods, one of which is given here. The surface of silicon wafer was initially coated with polyethylene oxide (PEO) to prevent the adsorption of DNA molecules and laser was applied for the designed region where adsorption of DNA is favored [19]. We use this work as our case study and Monte Carlo simulation of such heterogeneous adsorption is our focus in this study. The simulation detail is provided in the next section. The relative magnitudes of surface energies are adjusted and the corresponding monolayer structures will be generated and presented together with their radial distribution functions.

## 2. Simulation Methods

**Figure 1:**
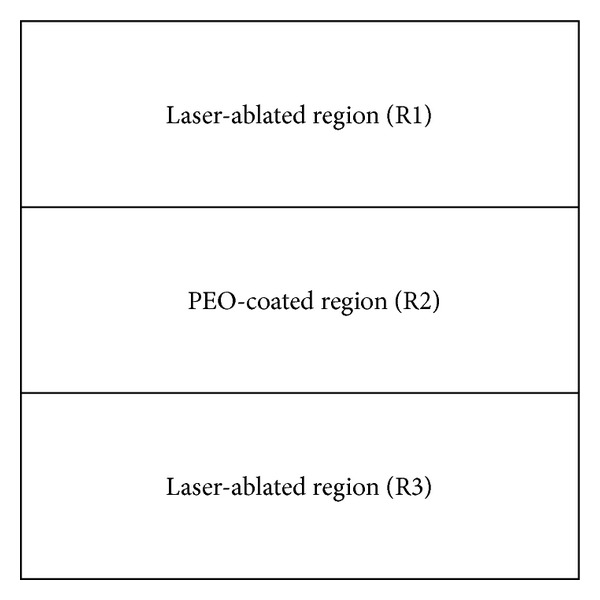
The specified pattern consisting of ablated regions (R1) and (R3) and PEO-covered region (R2) for DNA adsorption.

Adamczyk et al. [20] investigated mechanisms of nanoparticle and bioparticle deposition in which heterogeneous adsorption was partly discussed. Their method is to generate the adsorption sites on the surface first by assuming the sites as very small particles compared with deposited particles. If the overlap between a site particle and a deposited particle occurs, the adsorption is successful and the deposited particle is adsorbed at that position forever. This method is appropriate for adsorption at random or specific sites on the surface. However, in our framework of patterned heterogeneous surface, a new method is more appropriate and is described here. We choose to study the pattern of the treated surface as shown in Figure 1. According to [19], PEO was removed by laser from ablated regions (R1 and R3) and region at the center was untreated (PEO-covered) (R2).

Monte Carlo simulations were performed to collect the statistical data of the radial distribution function and generate typical configurations for analysis. The calculation of the radial distribution function *g*(*r*_*i*_ + Δ*r*/2) is based on a histogram for small increments of width Δ*r*. The averaged distribution function is then trivially obtained as the ratio between number of pairs *N*_*i*_ collected from *n* configurations, at the separation ranging from *r*_*i*_ and *r*_*i*_ + Δ*r* from the average spherical particle to the corresponding number in a system of ideal-gas or randomly placed particles. The function could be expressed as

(1) $$\begin{array}{c} g\left(r_{i}+\frac{\Delta r}{2}\right)=\frac{N_{i}}{\pi r_{i}\Delta r\rho Nn}, \end{array}$$

where *N* is the total number of particles in the system and *ρ* is the number density of the system.

The adsorbed particles or proteins are assumed to be spherical particles which are added one by one onto a 20*d* × 20*d* surface, where *d* is the hard-core diameter of a particle. The detail of adsorption is according to SQ model [12]. If an added particle overlaps with previously quenched ones, it is removed and a new addition is attempted until the insertion is successful. The particle is then moved randomly on the surface under the overall effect of pair interactions represented by the modified triangular-well potential expressed in (2) until it gets equilibrated and fixed in place. The new insertion is then attempted in this manner until the reduced number density of particles on the surface, ρ* = *ρd* ^2^, reaches 0.3:

(2) $$\begin{array}{c} u\left(r_{ij}\right)=\{\begin{array}{cc} \infty , & r_{ij}<d,\\ -\frac{\varepsilon }{U}\left(\frac{\lambda d-r_{ij}}{\lambda d-d}\right), & d\leq r_{ij}\leq \lambda d,\\ 0, & r_{ij}>\lambda d. \end{array} \end{array}$$

Here, *u*(*r*_*ij*_) is the pair potential between particle *i* and particle *j* which are *r*_*ij*_ apart. We choose *λ* = 1.5, or equivalently the well width is equal to 0.5*d*, where *d* is the particle diameter and *ε* is the depth of the energy well. It is noted that *ε* is scaled with *U*, which is a factor to be adjusted. This factor is equal to *U*_1_ when the particles are moving on R1 and R3 areas and equal to *U*_2_ on R2 region.

To simulate surface diffusion, Markov rule is applied to generate a random walk such that the probability of visiting a particular point **r** is proportional to the particle's Boltzmann factor at that location, *e* ^−*βU*(**r**)^. Therefore, the move from **r** to **r**′ is accepted with probability,

(3) $$\begin{array}{c} P_{\text{move}}=\min\left(1,\exp\left\{-\beta \left[U\left(r^{\prime }\right)-U\left(r\right)\right]\right\}\right), \end{array}$$

where *U*(**r**) is the total energy which is from the summation of all pair potential functions *u*_*ij*_ in the system. *β* = 1/*kT*, where *k* is the Boltzmann constant and *T* is the absolute temperature. The reduced temperature, T* = *kT*/*ε*, which reflects the strength of attractive interactions, is arbitrarily set at 1.0. A particle performs a minimum of 3000 moves with a maximum displacement of 0.5*d* before it is quenched in place.

In all cases, the periodic boundary conditions are employed. The final results are averaged over at least 100 realizations. The effect of finite size was also checked by comparing the results with ones performed on 1000*d* ^2^ simulated surfaces. It was found that the differences were less than 0.3%. The results did not significantly depend on the system size because the pair interaction is short ranged. The results are provided in the following section when the values of *U*_1_ and *U*_2_ were adjusted.

## 3. Results and Discussion

**Figure 2:**
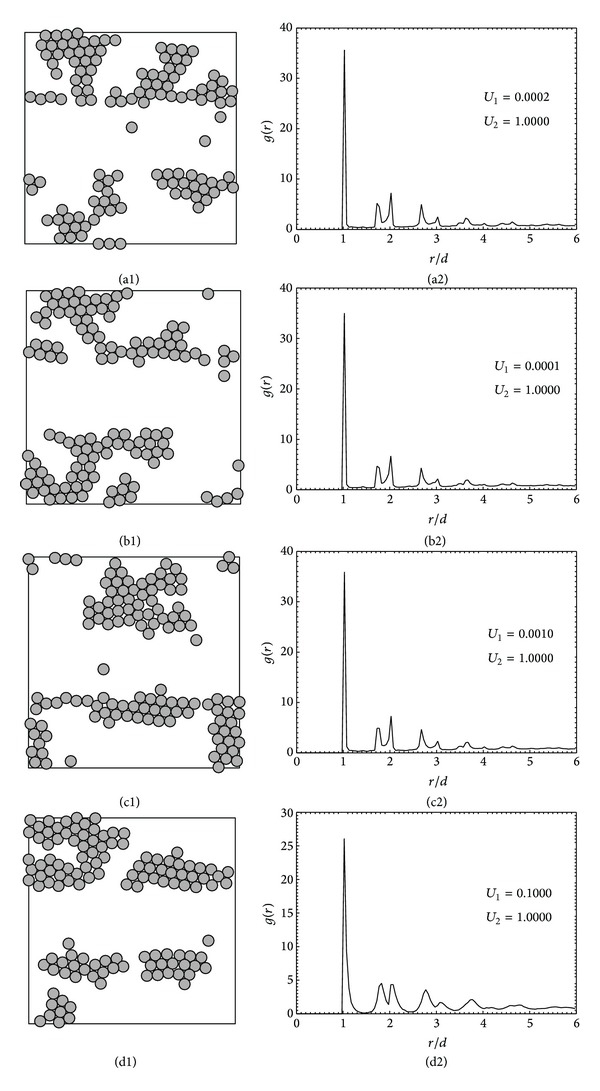
The monolayer structures (a1)–(d1) and the corresponding radial distribution functions (a2)–(d2) obtained for heterogeneous adsorption with varying ratio of surface energies of different regions: the ratio *U*_1_/*U*_2_ is 0.0002, 0.0001, 0.001, and 0.1.

**Figure 3:**
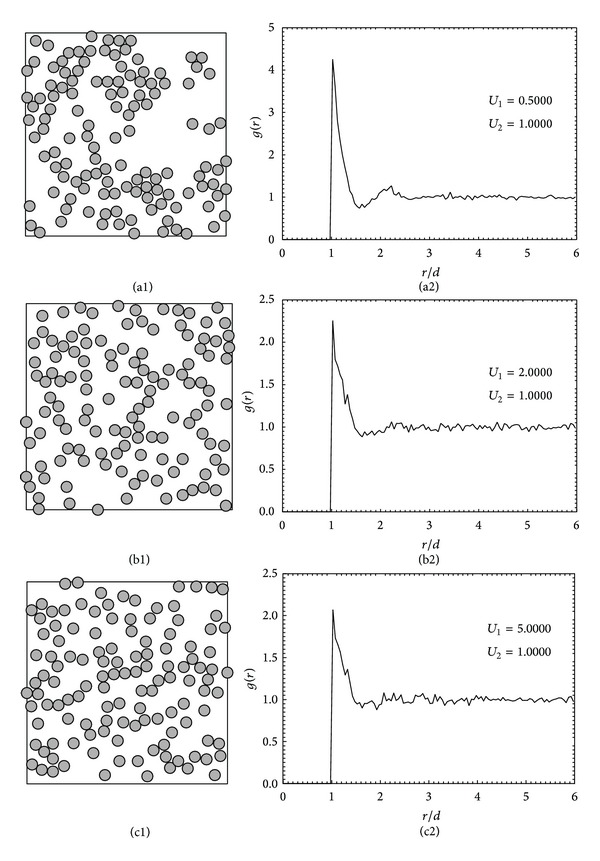
The monolayer structures (a1)–(c1) and the corresponding radial distribution functions (a2)–(c2) obtained for homogeneous adsorption with varying ratio of surface energies of different regions: the ratio *U*_1_/*U*_2_ is 0.5, 2.0, and 5.0.

When keeping *U*_2_ equal to 1.0 and varying *U*_1_ to be 0.0002, 0.0001, 0.001, and 0.1, the results are shown in Figure 2, while Figure 3 shows the results for varying *U*_1_ to be 0.5, 2.0, and 5.0. The attractive interaction of particles in Figure 2 is so strong that particles, after diffusing on the surface for quite some time, could aggregate and form clusters or two-dimensional islands on the ablated areas (R1 and R3), which resemble what was found in the experiments [19], in which DNA could be immobilized on ablated areas but could not be attached to PEO-coated surface. However, in some simulated configurations, a few particles are seen on R2 area. This should be from the possibility that 3000 moves may not be enough for the whole structure to reach a minimum energy which is a common situation in irreversible adsorption where the structure may not reach the equilibrium state.

The structures in Figures 2(a1)–2(d1) are somewhat similar to one another but the difference could be observed for the corresponding radial distribution functions, *g*(*r*), shown in Figures 2(a2)–2(d2). It is seen that the first peak of *g*(*r*) is lower when the ratio of   *U*_1_/*U*_2_ is higher because higher values of *U*_1_ mean weaker attractive interactions among particles. However, for the highest ratio of 0.1, the separation of adsorption zone could still be clearly seen. Other prominent peaks are also seen in Figure 2 representing that particles are packed in the ordered structure even though the degree of ordering is less at farther distance from the center particle.

Compared to those in Figure 2, the structures in Figures 3(a1)–3(c1) are disordered when the ratio of   *U*_1_/*U*_2_ was chosen to be higher than 0.1. The comparison of *g*(*r*) is given in Figures 3(a2)–3(c2). The probability of adsorption on each zone is in fact equal if the ratio is adjusted to 1.0 when there is no energetic heterogeneity on the surface. Therefore, when the ratio is 0.5, the probability for adsorption on R1 and R3 regions is still slightly greater than R2 as seen in the sample configuration. For the ratio of *U*_1_/*U*_2_ equal to 2.0 and 5.0, the structures resemble those obtained from random sequential adsorption (RSA) or sequential quenching (SQ) where the attractive forces between the adsorbed particles are weak and the surface is energetically homogeneous. Therefore, the relative magnitude of *U*_1_/*U*_2_ could be simply adjusted to represent both the heterogeneous and the homogeneous adsorption.

Basically, the total energy of the whole system is the summation of all types of interactions as described in

(4) $$\begin{array}{c} U_{\text{total}}=\underset{ij}{\sum }u_{ij}+\underset{i}{\sum }u_{i,\text{air}}+\underset{i}{\sum }u_{i,\text{surface}}+\underset{}{\sum }u_{\text{surface},\text{air}}, \end{array}$$

where *u*_*ij*_ is the potential energy between two particles, *i* and *j*, *u*_*i*,air_ is the energy between particle *i* and the air, *u*_*i*,surface_ is the energy between particle *i* and the surface, and *u*_surface,air_ is the energy between the surface and the air. In the adsorption of particles on heterogeneous surfaces, the interaction between particles and different surface is different and other terms could be considered the same and the equation is simplified to

(5) $$\begin{array}{c} U_{\text{total}}=a_{i}\underset{ij}{\sum }u_{i,\text{surface}}. \end{array}$$

Here, *a*_*i*_ is a factor greater than unity. Therefore, the relative magnitude of total energy for adsorption on energetically heterogeneous surface could be represented by the ratio

(6) $$\begin{array}{c} \frac{U_{\text{total},\text{R}1}}{U_{\text{total},\text{R}2}}=\frac{a_{1}\sum_{i,\text{R}1}u_{i,\text{surface}}}{a_{2}\sum_{i,\text{R}2}u_{i,\text{surface}}}. \end{array}$$

In this study, the effect of surface heterogeneity was incorporated into the pair potential function instead of separating both interactions. Therefore, the ratio may be approximated as

(7) $$\begin{array}{c} \frac{U_{\text{total},\text{R}1}}{U_{\text{total},\text{R}2}}=\frac{N_{\text{pair}}\varepsilon /U_{1}}{N_{\text{pair}}\varepsilon /U_{2}}=\frac{U_{2}}{U_{1}}. \end{array}$$

Here, *N*_pair_ is the number of pairs. As seen in (7), the preferable zone for adsorption has to have high surface energy. For this modified triangular-well potential, particles would rather be adsorbed on R1 and R3 regions than on R2 region when the relative magnitude of *U*_1_/*U*_2_ is less than or equal to 0.1. This is equivalent to *U*_2_/*U*_1_ equal 10, which means that surface energy of R1 has to be 10 times higher than that of R2 in order for particles to be selectively adsorbed.

There are experimental results showing the effect of surface energy on adsorption preference. Satriano et al. [21] investigated cell adhesion on surfaces of two different polymers, which were poly(hydroxymethylsiloxane) (PHMS) and poly(ethylene terephthalate) (PET), before and after treating surfaces with ion beam irradiation. It was shown that after surface treatment, there was an increase in number of cells attached to PHMS from 2.5 × 10^3^ to 2.5 × 10^4^ cell/cm^2^, while there was a decrease in number of cells attached on PET from 1.5 × 10^4^ to 1.25 × 10^4^ cell/cm^2^. The findings could be explained when considering the surface free energy. For PHMS, the energy increased from 25 to 50 mJ/m^2^, whereas for PET it was about the same of 40 mJ/m^2^. As suggested by thermodynamics, a system will undergo a change in order to decrease its free energy, which is also true for this case where the surface energy was minimized after particles were adsorbed on the surface.

Another experiment also described the influence of surface energy on adsorption. Rick and Chou [22] performed the experiments on protein adsorption. When adding lysozyme in a polymer to construct a template for protein adsorption and testing with lysozyme itself, the change of enthalpy was found to be 17.93 mJ. When tested with myoglobin, cytochrome C, and bovine albumin, the change of enthalpy was 40%, 45%, and 10% of that for lysozyme. The results of enthalpy change implied that the most favorable adsorption on lysozyme-template polymer was for lysozyme itself. For 1 gram of this polymeric adsorbent, the adsorption ability for lysozyme, myoglobin, cytochrome C, and bovine albumin was 0.375, 0.4, and 0.1, respectively, in good accord with the enthalpy change.

**Figure 4:**
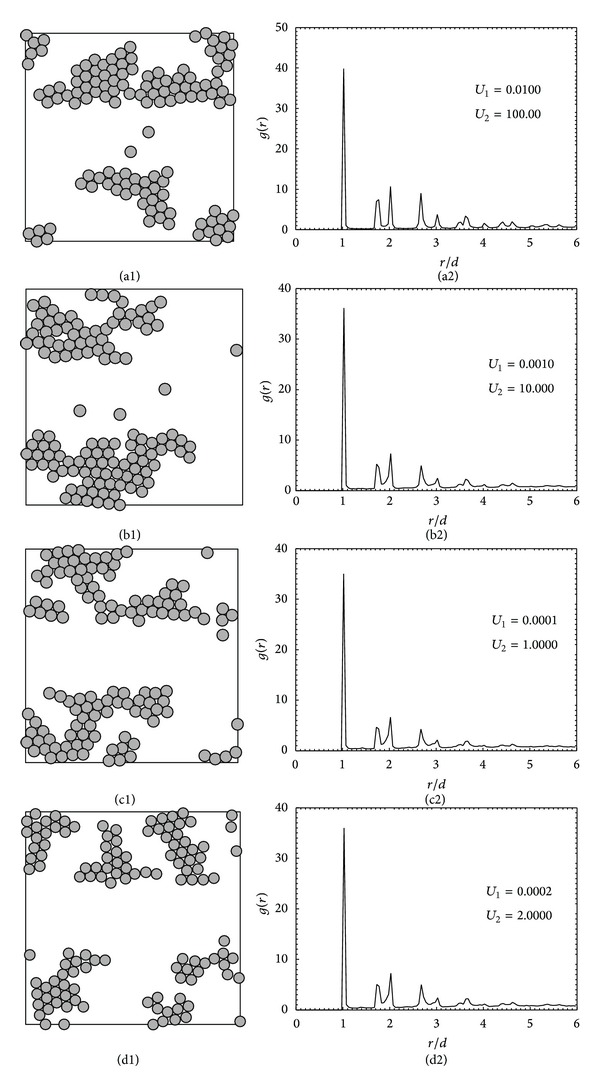
The monolayer structures (a1)–(d1) and the corresponding radial distribution functions (a2)–(d2) obtained for heterogeneous adsorption with a fixed ratio of surface energies (*U*_1_/*U*_2_) equal to 0.0001.

As discussed earlier, the effect of varying the ratio of *U*_1_/*U*_2_ is clearly observed in both Figures 2 and 3. Moreover, it is interesting to compare the results when fixing the ratio of *U*_1_/*U*_2_ constant at a high value of 10,000 and varying the values of *U*_1_ and *U*_2_ according to this ratio. The high value guarantees that the interactions of all particles are so strong that the structure is a compact monolayer. The results for *U*_1_ : *U*_2_ equal to 0.01 : 100, 0.001 : 10, 0.0001 : 1.0, and 0.0002 : 2.0 are shown in Figure 4. Figures 4(a1)–4(d1) show the simulated configurations, while Figures 4(a2)–4(d2) display their corresponding radial distribution functions. The configurations of all cases look similar and the radial distribution functions, *g*(*r*), are also similar. It seems that the obtained structures resemble one another when the ratio of *U*_1_/*U*_2_ is the same.

## 4. Conclusions

The modified triangular-well potential was used successfully to represent the incorporation of both particle-particle interaction and particle-surface interaction in this study. The system temperature, the well width of attractive part of the potential, and the number density were fixed, while the value of parameter representing the relative magnitude of surface energy was varied. The transition from homogeneous to heterogeneous adsorption was seen when the ratio of surface energies has to be greater than some value, which in this case is 10. The method is convenient to simulate the effect of energetically heterogeneous surface on adsorption of particles or bioparticles such as proteins. A variety of adsorption conditions could be investigated further in more detail by varying the system temperature reflecting the strength of interaction of particles as well as the attractive well width reflecting the attractive range of interaction. These parameters could be adjusted to simulate the actual protein adsorption.
